# Collectanea Medica, Consisting of Anecdotes, Facts, Extracts, Illustrations, Queries, Suggestions, &c.

**Published:** 1817-04

**Authors:** 


### COLLECTANEA MEDICA,

CONSISTING OF

### ANECDOTES, FACTS, EXTRACTS, ILLUSTRATIONS, QUERIES, SUGGESTIONS, &c.

*Observations on the Structure of the Brain, comprising an Estimate of the Claims of Drs. Gall and Spurzheim to Discovery in the Anatomy of that Organ.* By JOHN GORDON, M.D. F.R.S. Ed. Lecturer on Anatomy and Surgery, and on the Institutes of Medicine, Member of the Royal Col. of Surgeons in Edin. and one of the Surgeons of the Royal Infirmary. 8vo. Ed. Blackwood, 1817.—pp. 215.

*Examination of the Objections made in Britain against the Doctrines of Gall and Spurzheim.* By J. G. SPURZHEIM, M.D. 8vo. Edin. and Lond. Baldwin and Co.—pp. 87.

NOTHING is so destructive to the improvement of science as the chilling blast of ridicule or invective which every improver expects to receive. Unfortunately, nothing is so easy as to find fault, and, to young writers, nothing so pleasant as ridicule! The boy *modo custode remoto* thinks nothing so charming as to bask with his newly unfettered limbs in the sunshine of liberty, and to retort on his elders all the drudgery to which he has been, as he conceives, so unreasonably confined.

The more noble the game, the greater the merit. Thus, the name of Hunter, so long celebrated throughout Europe, was, in the Edinburgh Review, attacked with most unhallowed indecency, by one who seemed to boast that he was young enough to be the grandson of Abernethy,* the eulogist of his venerable master. This was not enough, but the strippling must threaten in a future number to attack names which had passed the ordeal of the first anatomists in an university always celebrated for the number and accuracy of its dissections. All this might have been pardoned, had the juvenile critic apologized for, instead of boasting of, his youth. But, whatever apologies his friends may think him entitled to on that score, nothing can ever excuse the conductors of the Review for permitting two such articles from such a source. It is, however, something in extenuation to find, that, from a conviction of their error, which it would become them to acknowledge, they have since declined mixing in a contest they cannot understand.

* "We have laid down of late, a plan of the strictest economy as to the expenditure of that portion of the very *quickly and powerfully mobile* substance which has fallen to our share, and we are not without hopes that it may thereby be made to last for three-score years to come: it is that plan, not to squander our youth."—*Extract from the Edinburgh Review of Mr. Abernethy's Lecture on Mr. Hunter's Theory.*

It

It is very true, as we shall see it presently remarked, that the French anatomists did not entirely agree with the whole of the new doctrine. It would, indeed, have been strange, if they had. Surely, the warmest votaries would scarcely require that men, some of whom have passed their seventieth year, should instantly adopt a science more new than the circulation of the blood in the days of Hervey. Their report, though cautious, was candid, and, compared with the coarseness with which the same characters have been treated in Britain, will be among the accusations of vulgarity so often pointed against us. A commission of the most distinguished members of the National Institute, in a Memoir signed by the illustrious Cuvier, declared that "the opinions of Gall and Spurzheim would not be controverted *in limine*, by those whose only wish is to be informed;" and, in several other parts, they admit, that "they have [Gall and Spurzheim] proved what was imperfectly described by some of the older anatomists, and, consequently disregarded by their successors; and that they are the first who have distinguished the two orders of fibres of the medullary matter of the two hemispheres," &c.

Such a report as this might have rendered us cautious on this side of the water. We might, at least, have recollected, that in this instance, the French anatomists sacrificed their national vanity, and submitted to be taught by Germans. It is not to be wondered if the founders of a new doctrine were dissatisfied with even this document in their favour. But we are scarcely authorized to say so much. There is rather reason to believe, that having so far gained the public opinion, their wish was, by further explanations, to satify every remaining doubt. As might have been expected, from one of these causes, they renewed their experiments, and gave the result with greater minuteness, and with a proper attention to the objections of such candid reporters.

Every one of our readers must be aware, that even demonstration on so nice a point is difficult, unless conducted under every possible advantage, and by persons in the constant habit of such examinations. Description, and even plates, though highly necessary for comprehending the subject, must be useless as matter of controversy. We have, therefore, made long selections from such passages as we found most interesting, most intelligible, and most likely to enable our readers to comprehend the various parts of the controversy.

The following is part of Dr. Gordon's introduction to his Observations.

"The interest I necessarily feel in the advancement of anatomical science, made it impossible for me to hear with indifference, that certain discoveries had been made, some years ago, in the anatomy of the brain, by two German physicians, Drs. Gall and Spurzheim. But, though the matter did not escape my notice, I had no opportunity of considering it with the attention its importance required, till I was occupied in preparing a description of the nervous system, for the first volume of my System of Anatomy.

 It

It then became necessary for me to examine, with all care, the writings of these gentlemen on the same subject, to repeat their dissections frequently, and to compare their descriptions and engravings strictly with nature. I will not pretend that this inquiry left me very doubtful concerning the accuracy of their observations. To a certain degree, however, I did suspend my judgment; as it was just possible that the appearances and structure which they claim the merit of having first displayed, had been invisible to me in consequence of some mistake in the method of dissection, or a want of expertness in the manipulations peculiar to these anatomists. It was with considerable satisfaction, therefore, that I learned Dr. Spurzheim's intention, after his arrival in this place, to give a demonstration of the brain. I have twice seen his dissections of this organ; but I am sorry to say, that, by these opportunities, the unfavourable estimate I had formed of his and Dr. Gall's labours, has been not removed, but confirmed. I cannot but believe, that their pretensions are yet as unfounded as they have been confidently advanced; and are, both in their results and in their principles, calculated to retard the progress of real knowledge.

"This opinion has been adopted after long and painful research; and I own it neither surprises me, nor (if I may be pardoned for so speaking) diminishes my confidence, that others, whose names are not unknown to the medical world, have expressed different sentiments. An intimate acquaintance with the structure of the brain is exceedingly difficult of acquisition; the organ is peculiarly delicate, and its parts are intricate and numerous. Fully proportioned, however, as the importance of this knowledge may be to the obstacles in the way of attaining it, in a physiological point of view, it is fortunately not of essential consequence in the practice of medicine. Diseases may be distinguished from each other, and the proper remedies applied to them, without it; and hence it happens, that a study at once arduous, and in a great measure unnecessary, is easily relinquished; and that skilful and eminent practitioners are satisfied, and justly so, with a general view of the structure of this organ. The information, too, demanded by the profession, cannot fail to influence the learning and the lessons of its instructors; who have little inducement to possess, as they have none to impart, more knowledge than they are required or expected to convey. It is neither unaccountable nor unreasonable, therefore, that the anatomy of the brain should be imperfectly known to many, who are distinguished in teaching and practising the various branches of the medical art.

"These circumstances are undoubtedly favourable to the progress of any hypothesis respecting the structure of this part of the human frame. Provided its author have the prudence to found it on some established truths, and the dexterity to represent it as the means of explaining certain phenomena hitherto inexplicable, he may often venture, with great prospect of success, to bring to its support facts not observable in nature, and not the parents, but the the offspring of his theory. The persons he addresses, whose early studies (never, perhaps, in this department complete) have much faded from their recollection, and whom the duties of an active and toilsome profession prevent from yielding the attention requisite for mature judgment on such a subject, are apt to acquiesce in any statements having an air of plausibility, and asserted with confidence. Without time or opportunity for experiment or observation, they are willing to compromise; and to receive that as correct, which looks as if it might be so.

"In some such way as this alone, can I account for the support, with which the alleged discoveries of Drs. Gall and Spurzheim are said, in several places, to have been honoured. For I am quite persuaded that no one can consult the history of the science, and afterwards appeal to nature, without being convinced, that such of their statements as are peculiar to them, owe all their plausibility to inaccuracy, to the neglect of some appearances, and to the imagining of others; and that the descriptions which they have derived from the writings of other anatomists, and which they have sometimes modified but never improved in the transference, make up the whole portion of truth which this new system comprehends.

"Entertaining this opinion,—satisfied that it must be sanctioned by all attentive inquirers,—and anxious to check, as far as I may, errors of such magnitude in anatomy, I have been encouraged to lay the following pages before the profession and the public. Little, perhaps, can be said that would not readily occur to those, who attempt to verify, by dissection, the descriptions of Drs. Gall and Spurzheim. But as this test is not always convenient, and as the works of other anatomists, whose labours must not be forgotten in estimating the merits of their successors of this day, are not always accessible or easy of reference, it was represented to me, that the result of my inquiries would not be unacceptable to those who, with smaller opportunity or occasion of study, wished to have some materials for forming their opinion on a scientific question of such interest.

"On a subject of this kind, it is not easy, without demonstration, to be always quite intelligible. The force of mere authorities is likely to be sometimes overlooked by the reader; and it is natural to expect, that in the end a good deal may still appear as disputed assertion. But, notwithstanding these difficulties, the late claims to discovery will still, I trust, be sufficiently refuted. They will always be found more groundless the more narrowly they are inspected; and my pains cannot be altogether without reward, if they prove effectual in promoting investigation. I have only to add, that nothing could have engaged me to publish the present remarks, but a strong anxiety for the progress of medical knowledge, and a deep concern for the reputation of a medical school, which was indebted to anatomy for its first celebrity throughout Europe.

"The most complete summary of the doctrines which it is my object object to examine, is to be found in the article *Cerveau,* in the 4th volume of the Dictionnaire des Sciences Medicales, published at Paris in the year 1813. It is written by Drs. Gall and Spurzheim themselves, and bears their signature; and, as it is the latest dissertation which they have composed on the subject, it may be presumed to contain the expression of their most matured opinions. This essay I have inserted *verbatim* in the Appendix; and it is my intention to refer to it constantly, in the following pages. Two advantages will result from this. In the first place, it will enable my readers to perceive whether or not I have, on all occasions, correctly interpreted the meaning of the descriptions which are the object of my criticism; and, secondly, it will afford those, who have no other means of knowledge, an opportunity of acquiring a perfect idea of the claims which Drs. Gall and Spurzheim have brought forward. In this last respect I cannot refrain from remarking, that I believe the Appendix to be particularly necessary; for I am firmly persuaded, that the number of persons is exceedingly small, in this country at least, who have any conception either of the nature or the extent of the discoveries alleged to have been made by the authors of this new system. Their work in quarto,* of which the first volume was published at Paris in 1810, is too expensive for extensive circulation, and much too tedious for general perusal, even were it more accessible; while the abstract of their anatomy of the brain prefixed by Dr. Spurzheim to his English book on Physiognomy,† is, in several essential points, imperfect.

"In the year 1808, the Institute of France appointed a Committee of its members,‡ to examine and report on a Memoir which had been presented to it by Drs. Gall and Spurzheim, containing an account of their anatomical researches and alleged discoveries. So little satisfied, however, were these anatomists with the report,§ which was returned, and with the small measure of praise granted to them by the Commissioners, that, in the following year, they published a quarto volume, consisting of 274 pages,|| wholly occupied with a criticism on the decision of the Committee; and in which, with the exception of a single article, the sentiments of the Commissioners implying partial approbation, as well as those ex-

"* Anatomie et Physiologie du Systeme Nerveux en General, et du Cerveau en particulier, &c. Prem. Vol. 4to. Paris; avec 17 planches folio.

"† The Physiognomical System of Drs. Gall and Spurzheim, &c. by J. G. Spurzheim, M.D. 8vo. 2d edit. Lond. 1815.

"‡ The Committee consisted of Tenon, Portal, Sabatier, Pinel, and Cuvier.

"§ There is a translation of this Report in Edin. Med. and Surg. Journ. for January, 1809.

"|| This volume is entitled 'Recherches sur le Systeme Nerveux en General et sur celui du Cerveux en particulier.' Paris, 1809.

pressing pressing direct dissent, are alike impugned and protested against. To this work, also, I shall sometimes have occasion to refer, in the course of the following pages.*

"In the three first paragraphs of the Appendix, it will be seen that Drs. Gall and Spurzheim, after stating various objections to the methods of investigating and describing the brain which have hitherto prevailed, claim to themselves the merit of having discovered a new mode of dissecting this organ, viz. of commencing the examination of each part from its first origin, and of following the course and direction of its fibres *by scraping* (en raclant). I propose, however, to reserve the particular examination of these criticisms, and of the claim which follows them, till the conclusion of the present pamphlet.

"I have, therefore, in the first place, to direct the attention of my reader to the opinions of Drs. Gall and Spurzheim respecting the structure of the *White* and *Grey Substance* of the brain in general. I shall then consider the two *Systems of Fibres* which they profess to have discovered in this organ. And, lastly, I propose to offer a few remarks on the *Engravings* of the brain which accompany their large work."

We now offer Dr. Spurzheim's Introduction.

"Discussions, properly conducted, are of great utility. For that reason, I am always ready to examine every objection against our doctrines. But I am sorry to observe, that scientific pursuits are so often degraded by selfish passions and the spirit of party;—that literary publications are employed for the purposes of calumny and detraction;—that invectives are used instead of arguments;—and that, by praising friends and blaming rivals, the progress of the arts and sciences, and the improvement of man, are mightily retarded.

"Such behaviour I will never imitate; nay, the illiberal and uncandid manner in which some British Reviews have taken up our investigations, has hitherto prevented me from attempting justification. As, however, many persons have no inclination, and a greater number no time, for comparing the original works with the reports of the critics; and, as in science the majority of readers believe, without examining for themselves, I cannot entirely avoid controversy. We have never published a separate answer to single pamphlets, but merely considered the objections in our Lectures or in our Works, when treating of the respective objects. Our maxim is, never to fight with darkness, but to endeavour to bring light.

"I am now to submit to the public some observations on the

"* It is amusing, notwithstanding the existence of this work, to hear the Committee of the French Institute occasionally named as supporters of the whole anatomical doctrines of Drs. Gall and Spurzheim."—Who has ever named them as such: see our introductory remarks to this article.—Edit.

objections objections of our principal antagonists in Great Britain, confining myself to the points in question, and depending on the moral sense, the judgment and observation, of my readers. In short and concise expressions, I will state the real object of our inquiries, and the true import of our propositions, and then compare the interpretations of the chief Reviews, especially of the literary gospel of Edinburgh. At the same time I will mention an antagonist, who was at first anonymous, but did not long conceal himself; who then appeared as an author on the structure of the brain, and at last as a historian of the anatomy of that organ.

"The Edinburgh Reviewer speaks (No. 49, p. 229) of 'a conscientious discharge of duty on this occasion;' it therefore is right to name him accordingly. The author of the Treatise on the Brain, in a pamphlet, asserts, that the anatomy of the brain is imperfectly known, even to the distinguished teachers of the medical art in Edinburgh; that the persons I have addressed, never, perhaps, have completed their studies in this department (p. 4); that I have shown the corpus dentatum to spectators, most of whom had never seen it before, and not one of whom had rendered himself familiar with its appearance by dissection (p. 43*). Hence, if there be only one person in Edinburgh who can judge of the appearances of brown and white, he deserves the name of anatomist *par excellence.* As, in his Treatise on the Brain, he states, (Pref. ix.) that he has scrupulously avoided the introduction of any physiological matter; and as in the pamphlet he maintains, that the anatomy of the brain, *in a physiological point of view*, is fortunately not of essential consequence in the practice of medicine (p. 3), I will style him a mechanical dissector. Another name which he merits is that of historian, because he has compiled

"* As the reader may wish to know who my auditors were, I will mention the names of some gentlemen. At the first demonstration were present, Dr. John Thomson, Prof. Regius of Military Surgery; Dr. Barclay, Lecturer on Anatomy and Surgery; Dr. Duncan, junior, Prof. of Medical Jurisprudence; Drs. Emery and Irvin, of the Military Staff. At the second, were Dr. Rutherford, Prof. of Botany; Dr. Home, Prof. of Materia Medica; Dr. Thomas Brown, Prof. of Moral Philosophy; Prof. Jamieson; Drs. Farquharson, Dewar, Sanders, Anderson, and a great number of professional gentleman. At the Physical Society I gave the demonstration in presence of Dr. Monro, junior, Prof. of Anatomy and Surgery; Drs. Rutherford, Barclay, and Sanders; Mr. Bryce, President of the College of Surgeons; Mr. George Bell, and a numerous audience of medical gentlemen. Since that time, I have often repeated these demonstrations in private parties, and always to the satisfaction of the spectators. *It is worthy of notice, that the essential point alluded to was, whether there is brown matter in the corpus dentatum? This had been denied by the Edinburgh Review*, p. 264."

 facts, facts, excellent indeed—concerning the history of the anatomy of the brain.

"Every one will perceive, that our adversaries are very witty men. They deal extensively in the ridiculous; and, when they have leisure to become serious, they speak of the motives and *dangerous consequences* of our inquiries; but their generous minds need not be apprehensive, since they declare our doctrines 'incredible and disgraceful nonsense, absurd theories, trash, and despicable trumpery.' If that is the case, while, as they admit, we make proselytes, they have, indeed, very little confidence in the discernment of their countrymen. Why do they not rather listen to our constant declaration, that one fact, well observed, is more decisive to us than a thousand opinions and all the metaphysical reasoning of the schools; and that facts alone can expel such intruders as our doctrines?

"These observations will be divided into three chapters. The first will contain Anatomical, the second Physiological, and the third Philosophical, considerations."

By this latter quotation it would appear that the article to which we objected in the Edinburgh Review, (the critique on Mr. Abernethy's Oration,) and in which Mr. Hunter was so ignorantly traduced, was written by Dr. Gordon. This must be an error of Dr. Spurzheim's, arising, we presume, from a want of acquaintance with our language, and particularly our academical language. In most parts of the continent, a College, which, with us, usually implies an university, is a term applied to a school, and a professor is a name given to every one who has acquired a Doctor's degree, inasmuch as he is presumed capable of teaching. This, or some other cause, must have led the German physician into the error of supposing it possible, because the Edinburgh Reviewers may have indulged their indolence or partiality to a juvenile prattler, that therefore, the author of such a paper should find an audience in a metropolis, and in the seat of an university, or that he should venture to teach anatomy, surgery, and the institutes of medicine. We have already expressed our wish that some little knowledge should be required of teachers in London; but, though the obstetric teacher to whom we refer evinces his ignorance of the anatomy of an organ, and its appendages, the knowledge of which should precede all scientific inductions, yet, compared with the anonymous writer who ventured to attack Hunter and Abernethy, he may at least lay claim to comparative modesty.

Our readers being, we presume, almost all medical, must be aware of the impossibility of our entering into the controversy, either as a matter of dissection, of physiology, or of philosophy in general. Of dissection, because no persons but practical anatomists can retain the figure of the various parts of the brain in their mind; and when the two parties describe colour and configuration differently, we shall not be expected to decide between them.

them. We may, however, take the liberty to remark, that the Edinburgh Reviewer is well known to be a lawyer; and the person employed in, or permitted, to review an article on this delicate organ, tells us he is a boy. In the Quarterly Review we are informed that this article was left for the criticism of a Clergyman. In this manner the two reviews, which claim the highest rank by the partisans on both sides, are conducted. The Edinburgh Review has always disgraced itself whenever it has undertaken a medical article, and, to do justice to the conductor, it must be admitted that he became at last so much ashamed of his medical reviewers, as to refuse any more articles of that kind, till, in an evil hour, he permitted this youth to sport with such names as Hunter, Abernethy, Gall, and Spurzheim.

The Quarterly Reviewers have shewn much more decency in their medical articles. But there seems in them a most unaccountable notion that opinions the most venerable, and which their best friends will be careful not to encumber with unnecessary dogmas, stand in perpetual need of their support. We shall not, however, enlarge on a subject foreign to our profession, but conclude with an extract which contains quotations from both those works, so ample as to justify us in placing the whole before our readers, though extracted from only one of the parties.

"The Quarterly Reviewer, however, thought proper to tell its readers, 'Of course, one instance is very properly considered just as satisfactory an evidence that the conclusion is conformable to fact, as a hundred would be,' No. 25, p. 169. 'Even admitting this system of Drs. Gall and Spurzheim to be ever so plausible as an hypothesis, it cannot possibly derive any sort of evidence from experience. For the same reason, it is equally impossible to contradict it from experience,' p. 171. 'Even allowing, that the arguments of Drs. Gall and Spurzheim, instead of being *sheer nonsense*, had been ever so ingenious and acute, still they could not throw the slightest probability upon the doctrine which they wish to establish, because that doctrine is *matter of fact*, and matter of fact never can be proved by reasoning *à priori*. Whether every protuberance upon the head be or be not the sign of some particular character of the mind, is clearly a question *of fact;* let it, therefore, be proved to be a fact, as all other facts are proved: in such a case, the explanation which Drs. Gall and Spurzheim propose, would at least have a fair claim to be heard,' p. 177. This, as Dr. Sp. observes, is another clear specimen that reviewers can criticise books without reading them! [It is most probable, the clerical gentleman, deceived by the boldness of his brother Edinburghers, supposed that all the manifestations exhibited in man and in other animals, were fabulous, and that the whole doctrine was supported by reasoning *à priori.*] From p. 262 to 271 in my book, our proceeding is quite differently described. I will copy only one sentence, p. 264. 'It is known that, in general, physical truths improve in proportion as observations are repeated. We continue,

 therefore, therefore, to multiply our observations, and as, in respect to several organs, the number of *these observations is immense, we consider the respective organs as established.* With regard to them, we must insist on our opinion, so long as from experience we are not convinced of the contrary. Several organs, however, are still only probable, and others merely conjectural, requiring a greater number of observations, in order to be determined with the same degree of certainty as those which are supported by the most satisfactory proofs.'

"The Edinburgh Reviewer, with respect to our proceeding, made 'some effort, and briefly observed, that not one of our assertions is true, and that not one step of our reasoning is correct,' p. 252. 'Can it be possible,' asks the philosopher, 'that the great Drs. Gall and Spurzheim have not observed, in the course of their multifarious inquiries into nature, that phenomena may coincide, without being related to each other as cause and effect? Were it established that all great mathematicians had black eyes, and all poets blue ones, would any sensible man, from this alone, think of ascribing the mathematical talent, in the one case, or the poetical genius in the other, to the colour of the iris?' p. 247.

"Had this learned Reviewer also studied Chap. I. of Part III. of my book, he would have seen, that we are aware of the difference between coincidence and the relation of cause and effect to each other, and never lose sight of it; that we prove our assertions in the same way as any physical truth. If, however, an observer could shew, that only mathematicians have black eyes, and only poets blue ones; that every one who has black eyes, and no one but those, have mathematical talents, or that every one with blue eyes, and only those, are born poets; if he could repeat his observations in various countries; if he could compare the same talents through a series of animals, without finding an exception; if he could support his observations by other means which I have detailed in my book, he might establish a physiognomical sign, and challenge his opponents to shew the contrary. So we do. If, for instance, we speak of a sign of self-esteem, let us see that a man, the most prominent feature of whose character is composed of self-conceit, does not exhibit the sign on his head, and we give up all our observations with respect to this peculiar organ. In the same manner, and by no other means, each organ is to be refuted by one single exception well ascertained.

"It cannot be useless to call the attention of the reader to that method which the literary gospel of Edinburgh, No. 48, Art. x. p. 448, recommends as follows: 'Sir Everard Home's Essay not only possesses a proper method of investigation, but sets an example of it, and is entirely free from the nonsense which is so commonly and so copiously put forth in writings upon similar subjects.' Which is then the proper method of investigating the functions of the brain? This the reader does not learn from the Review, but he may learn it from the original paper, inserted in the Philosophical Transactions for the year 1814, part 2. Sir Everard

Home there tells us, 'The various attempts which have been made to procure accurate information respecting the functions that belong to individual portions of the human brain, having been attended with very little success, it has occurred to me, that were anatomical surgeons to collect in one view all the appearances they had met with in cases of injury to that organ, and the effects that such injuries produced upon its functions, a body of evidence might be formed that would materially advance this highly important investigation.' He then informs us, that he has brought together certain observations, 'stating them as so many experiments upon the brain, with the conclusions which tend to elucidate this particular injury.'"

In doing this, Dr. S. very justly observes, that Sir Everard has shewn the effects of injuries to the brain without attempting to elicit any physiological inductions.

"Sir Everard Home (continues Dr. S.) speaks in a manner as if no one before him had made similar observations. His kind Reviewer, however, shews, by his numerous quotations, that Sir Everard is mistaken. Indeed, every one who is but half acquainted with the history of the healthy and diseased state of the brain, knows, that many authors have related similar facts. Nay, we learn from them also, that similar injuries of the brain have often been observed without any perceptible derangement of the mind, or any apparent disease of automatic life.

"Hence this mode of proceeding is quite unfit for discovering the functions of the brain, and any hope from such a source is in vain. I support my opinion by the fruitless attempt of a great number of authors, and by the unsuccessfulness of Sir Everard Home himself. It is true, he speaks of a body of evidence which might be formed, and of conclusions which tend to elucidate this particular inquiry, but he has not drawn even one inference. In the various pathological affections of the brain, he has observed head-ach, giddiness, faintness, loss of memory, want of sleep, delirium, mania, depression of spirits, melancholy, apoplexy, idiotism, hissing noise in the ears, deafness, blindness, loss of speech, irregular pulse, stupor, the mouth drawn to one side, numbness of the arms and legs, spasms in the lower extremities, stumbling in walking, pain between the shoulders, nausea, retching, slow action of purgative medicines, vomiting, convulsions, &c. Is Sir Everard Home, inclined to draw the inference, that the brain is the organ of these symptoms, or of the states which are opposite to them? This is, I think, sufficient to shew an intelligent reader, that in this way we never shall be able to determine the peculiar functions of the cerebral parts;—that the Edinburgh Review, for praising such a paper, deserves no more credit with respect to the physiology than to the anatomy of the brain, and that these critics, as they believe in the existence of cases which are in contradiction to nature and reasoning, have still a great deal to learn before they can become competent judges.

"SECTION

"SECTION V.—As to the individual organs of the manifestations of the mind, the same Review states only, 'To enter on a particular refutation of them, would be to insult the understandings of our readers. Indeed, we will flatter the authors so far as to say, that their observations are of a nature to set criticism entirely at defiance. They are a collection of mere absurdities, without truth, connexion, or consistency; an incoherent rhapsody, which nothing could have induced any man to have presented to the public, under a pretence of instructing them, but absolute insanity, gross ignorance, and the most matchless assurance.'

"Such arms, however, will not repel stubborn facts. Our antagonists, it seems, find it more easy to blame than to study, or to deny than to observe. They have not even considered the meaning of the expressions by which we designate the various powers of the mind. The Quarterly Review, for instance, states that the name *Inhabitiveness*, which I give to the instinct of animals, to live in water or on dry land, in higher or lower regions, and so on; to that instinct which determines a young duck, as soon as it is hatched, to run towards the water, and the ptarmaghan to dwell at the tops of the mountains, &c. means 'a love of dwelling in elevated situations.' He explains *Secretiveness* by the love of stealing. The natural history of the two species of rats, the black and the brown, he found very ridiculous; and he thought to exclaim, 'Credat Judæus Appella!' would be sufficient to change the cerebral organization of these two species of rats. I, however, must continue to say, that the difference of the brains of both species is easily distinguished. My auditors will recollect to have seen it. Thus, I repeat, to incontestable facts alone I shall pay further attention.

"The only reasonable difficulty started against the possibility of distinguishing the organs at the lower part of the forehead, and behind the orbits, originates from the frontal sinus, and from the circumstance that the brain, situate behind the orbits, and between both hemispheres, does not reach the surface of the skull. As, however, I have stated this difficulty, and given our explanation, the Reviewer ought to have copied our answer, instead of saying, 'How could these gentlemen think so poorly of the eye-sight of their readers, as to imagine, that, by the aid of their beautiful engravings, they could fail to discover, that some of the prominences in the skull which they describe, are said to be caused by elevations and portions of the brain, which are not even in contact with the skull of these parts?' p. 253.

"I always show to my auditors the difference between the external bony crest, often erroneously called frontal sinus, and the elevation, which we consider as a greater development of the organ of locality. They will also recollect my demonstrating, that children, and young and adult persons, have no holes between the two tables of the skull at the forehead; and that the real frontal sinus occur only in old persons, or after chronic insanity, in general, when the brain is diminished in size. I will copy only one passage from from my book, in opposition to that of the Edinburgh Review. 'The cerebral parts, situated behind the orbits, require some exercise on the part of the physiognomist, in order to be exactly determined. Their development is discoverable from the position and configuration of the eyes, and from the circumference of the orbits. It is, therefore, necessary to examine, whether the eyeball is prominent or hidden in the orbit, or whether it is placed inward or outward. According to the position of the eye-ball we may judge whether the part of the brain which is situate against a corresponding part of the orbit, is more or less developed.

"It may be questioned, whether all organs reach the surface, so as to enable us to determine the organs of all faculties of the mind by the size and shape of the head? There are, indeed, many convolutions in the middle line of the brain between the two hemispheres; and there are also some others at the basis of the brain, and between the anterior and middle lobes, which, therefore, do not reach the surface of the skull; but it seems to me that a great part at least of every organ lies at the surface, and that, if one part of any organ be well developed, the whole participates of this development. The whole cerebellum does not touch the skull, yet it is possible to determine the size of the cerebellum, according to that part of it which reaches the surface. Accordingly, the cerebral parts, which are, as above noticed, situate in the middle line between the two hemispheres, seem to be proportionate to the superincumbent organs; at least I have always observed a proportion in the vertical direction, between these cerebral parts. In this way, it appears to be possible to determine all the organs, though the whole of their fibres do not terminate at the surface.' P. 237, 238.

"There remains still an idea to be corrected. In pointing out the functions of the cerebral parts, and in ascertaining that the size of the organs has some influence on the innate dispositions of the mind, we establish, in a certain degree, a physiognomical doctrine. This has been most erroneously represented by the conscientious reviewer, in saying, p. 250, 'The practical part of their doctrines, as it may be called, the physiognomy, craniology, or cranioscopy, the part which teaches us how to find out, by the shape of the head, whether a man loves his children or kills them; whether he steals or is very benevolent!'—We, however, continually maintain, that we never can speak of the actions of man; and, after having mentioned the title, Physiognomical System, I begin the introduction of my book, 'This system is commonly considered as one according to which it is possible to discover the particular actions of individuals: it is treated as an art of prognostication. Such, however, is not the aim of our inquiries; we never treat of determinate actions; we consider only the faculties man is endowed with, the organic parts by means of which these faculties are manifested, and the general indications which they present.'

"Thus, the more the reader will compare our works, and the reports reports given by our antagonists, and their and our opinions with nature, the more he will be enabled to decide of whom it may be said, 'Were they even to succeed in shaking off the suspicion of *mala fides,* which we apprehend is inseparably attached to their character, we should not hesitate to say, that we do not know any writers, who, with a conceit so truly ludicrous, and so impudent a contempt for the opinions and labours of others, are so utterly destitute of every qualification necessary for the conduct of a philosophical investigation.'—*Edinburgh Review,* No. 49, p. 228. [Well said, boy.]

"Chapter III.—*Philosophy.*—This chapter may be very short, since in this department our British antagonists confine themselves to general considerations. The logical study of the author in the Quarterly Review, No. 25, p. 165, is the most simple: he admits in the mind only one understanding, and in that one he seems defective. 'There is, (says he) no more solid reason for dividing understanding into faculties, than for dividing heat or light into faculties.' This comparison, however, of understanding with heat and light, is not very apt for simplicity, since neither has been proved to be a single substance. Besides, as one single understanding does not explain the phenomena of the mind, and as all other logicians found it necessary to adopt several powers, I leave him to make the best use of his one faculty, and proceed to other propositions.

"The Edinburgh Review, as to the faculties which we adopt in the human mind, says, p. 243, 'The ratiocination of Drs. Gall and Spurzheim is of the most difficult species to combat. Perhaps we might content ourselves with saying, that the whole doctrine of the thirty-three faculties to which the argument relates, is downright nonsense, and so put an end to the discussion at once;—but we shall take the liberty of substituting for the names of the thirty-three faculties, two very simple and intelligible terms, viz. *intellect* and *inclination.*'

"The reasoning, or rather dogmatic decision of a Reviewer, certainly will not repel stubborn facts. I, however, should like to know, why the conscientious Philosopher adopts intellect *and* inclination. May I suppose that he does so, because one or the other alone does not explain the phenomena of the mind? Indeed, there may be strong inclination without intellect. But is inclination always the same? Is, for instance, the inclination of the hen towards the young duck, hatched by her, the same with the inclination of the young duck towards the water? Is the inclination to calumny or respect, to concealment or candour, one and the same? In the same way, is intellect only one? In a boy who can repeat by heart whole pages after having read them once or twice, but cannot compare or distinguish two separate ideas, is the intellect the same as in another who judges with precision of various ideas, but cannot recollect by heart one page?"

"One of our ideas, viz. the introduction of consciousness, sometimes sometimes active and sometimes passive, in the five senses, puzzled the Edinburgh Reviewer (p. 241) a good deal. The difference, however, seems to have been observed at all times, since, in all languages, there are two sorts of signs to express it. In the English we say, I see (passive) and I look at (active); I hear (passive) and I listen (active); I feel (passive) and I touch (active), &c. In other words, consciousness is sometimes involuntary, sometimes voluntary.

"These, and other considerations, are too complex for the simple philosophy of the Reviewers. As our opinions are not attacked in the particulars, there is no occasion for my giving here a more detailed explanation. Those who are desirous of knowing our philosophical propositions, will find them in my work on Physiognomy. I have only to add, that, if the conscientious Reviewer has found in himself only intellect and inclination, I leave it to others to judge, whether they have found his intellect limited in judgment, and his inclination extensive in malevolence.

"*Conclusion.*—Considering the whole of the preceding statements, I may say, that I have done with those who arrogate the right of thinking and deciding for the rest of mankind; with those 'thorough partizans, who are thorough despisers of sincerity;' (*Edin. Review*, No. 53, p. 14); who will not allow the least credit to any one that has not their approbation; who anonymously calumniate and detract; who, in doing so, claim the merit of conscientiousness; who disguise, mistate, and misinterpret; who invent ridiculous monstrosities; who, in using the most vulgar language, speak of personal dignity and politeness; with beings who change assertions as it seems convenient; who do not understand the passages which they quote;—who, from different chapters, extract sentences, illustrating different propositions, and represent these their own fictions, as nonsensical and absurd conceptions of the author;—with such writers on the brain, who have nothing in view but minute mechanical differences of size and form, and shades of colour; who, however, cannot see brown substance in the pons Varolii;—who, as if there were not, from ancient times, absurd names enough, invent in the brain, cul-de-sacs, pits, grooves, mountains, wings, lobules, and so on;—who never consider the parts in connexion and relation, nay, create artificial separations;—who are attentive only to the mechanical appearances, and never think of the functions of the parts;—who believe, that a man can walk, and have voluntary motion of his legs, without spinal cord, can philosophize without brain;—who can assert, that physiological inquiries of the brain are of no use to the medical profession; who consider one brain and its parts as the standard of all other brains; who admit, that the brains of *men* have their full growth at seven years of age, and do not undergo any change afterwards; and with such historians, who affirm from erudite research, and as the result of many experiments, made under a variety of circumstances, that there is no foundation whatever for the supposition, that that the convolutions consist of two layers;—who maintain, that numerous unequivocal instances are on record, and are even occurring every day, in which large portions of the brain, nay, almost the whole, if not actually the whole of this organ, has been completely destroyed by the progress of hydrocephalus;—who hold this to be a fact just as certain as that there are many persons now alive whose legs have been removed by the knife of the surgeon;—and who at another time prove, that we are not the first who maintain that the brain exists in hydrocephalic heads, and that Reil could separate the convolutions in the middle line, after we had shewn to him that structure four years before;—who denies the possibility of demonstrating the two sets of fibres (diverging and converging);—who does not mention the two layers of the convolutions;—and who afterwards, as pamphleteer, asserts, that long ago these things were known,—that especially we have defrauded Reil, who published four years after we had shown him our anatomical discoveries, after we had demonstrated them in different countries, in the universities of Germany, Denmark, Holland, and in Paris, and after the publication of numerous extracts by our pupils;—who tells his readers, that his pamphlet owes its origin only to his strong anxiety for the progress of medical knowledge, and deep concern for the reputation of a medical school which was indebted to anatomy for its first celebrity throughout Europe, but who makes morbid dissections, even in very rare cases, in the manner I have witnessed and described above;—who in that very pamphlet accuses all anatomists, and almost all medical professors and teachers of Edinburgh, and every one of my auditors, as unfit to distinguish brown and white substance;—who, in his 'painful' compilation, forgets the Monros, who deserve to be mentioned as well as Malpighi and Mayer; a neglect the less excusable, as Monro was one of the chief founders of the celebrity of the medical school of Edinburgh."

*** As Dr. Spurzheim is now lecturing among us, we shall revert to this subject under the division of "Intelligence."

